# Genomic ancestry, diet and microbiomes of Upper Palaeolithic hunter-gatherers from San Teodoro cave

**DOI:** 10.1038/s42003-022-04190-2

**Published:** 2022-11-18

**Authors:** Gabriele Scorrano, Sofie Holtsmark Nielsen, Domenico Lo Vetro, Rikai Sawafuji, Meaghan Mackie, Ashot Margaryan, Anna K. Fotakis, Cristina Martínez-Labarga, Pier Francesco Fabbri, Morten E. Allentoft, Marialetizia Carra, Fabio Martini, Olga Rickards, Jesper V. Olsen, Mikkel Winther Pedersen, Enrico Cappellini, Martin Sikora

**Affiliations:** 1 Section for Evolutionary Genomics, Globe Institute, University of Copenhagen, Copenhagen, Denmark; 2 Lundbeck Foundation GeoGenetics Centre, Globe Institute, University of Copenhagen, Copenhagen, Denmark; 3 Dipartimento di Storia, Archeologia, Geografia, Arte e Spettacolo (SAGAS), University of Florence, Florence, Italy; 4 Museo e Istituto Fiorentino di Preistoria, Florence, Italy; 5 Novo Nordisk Foundation Center for Protein Research, Faculty of Health and Medical Sciences, University of Copenhagen, Copenhagen, Denmark; 6 Center for Evolutionary Hologenomics, University of Copenhagen, Copenhagen, Denmark; 7 Centro di Antropologia Molecolare per lo studio del DNA antico, Dipartimento di Biologia, University of Rome Tor Vergata, Rome, Italy; 8 Dipartimento di Beni Culturali, University of Salento, Lecce, Italy; 9 Trace and Environmental DNA (TrEnD) Laboratory, School of Molecular and Life Sciences, Curtin University, Perth, WA Australia; 10 DANTE: Diet and Ancient Technology Laboratory, Sapienza University of Rome, Rome, Italy

**Keywords:** Population genetics, Archaeology

## Abstract

Recent improvements in the analysis of ancient biomolecules from human remains and associated dental calculus have provided new insights into the prehistoric diet and genetic diversity of our species. Here we present a multi-omics study, integrating metagenomic and proteomic analyses of dental calculus, and human ancient DNA analysis of the petrous bones of two post-Last Glacial Maximum (LGM) individuals from San Teodoro cave (Italy), to reconstruct their lifestyle and the post-LGM resettlement of Europe. Our analyses show genetic homogeneity in Sicily during the Palaeolithic, representing a hitherto unknown Italian genetic lineage within the previously identified Villabruna cluster. We argue that this lineage took refuge in Italy during the LGM, followed by a subsequent spread to central-western Europe. Analysis of dental calculus showed a diet rich in animal proteins which is also reflected on the oral microbiome composition. Our results demonstrate the power of this approach in the study of prehistoric humans and will enable future research to reach a more holistic understanding of the population dynamics and ecology.

## Abstract (web summary)

A combined ancient genomic, metagenomic, and paleoproteomic analysis reveals lifestyle and dietary information of Upper Palaeolithic huntergatherers from San Teodoro cave in Sicily, Italy.

## Introduction

In recent years, improvement of ancient DNA (aDNA) methods has given unprecedented insights into the past population dynamics of our species^1,2^. To date, most aDNA research focus on the dispersal of anatomically modern humans across the world, exploring the history of migration routes and admixture events which shaped extant human genetic variability. Recent studies have clarified the peopling of Western Eurasia during the Upper Palaeolithic after modern humans migrated out of Africa^1^. While the earliest people arriving in Europe around 45 kya appear not to have contributed ancestry to later groups^2^; the genome of an early European individual from Kostenki 14, dated to around 37 kya, demonstrated that the ancestral European gene pool was already established by that time^3^.

Analyses of genomic data from a larger set of pre-Neolithic Europeans documented complex population structure among early Europeans, involving multiple deeply diverged lineages. Among the identified pre- and post-Last Glacial Maximum (LGM) genetic clusters, two lineages, El Mirón and Villabruna^3^, share the highest number of alleles with present-day Europeans. Both lineages were widespread in Europe during the major warming period after the LGM, the Bølling-Allerød interstadial^4^. El Mirón (in Europe around 19–14 kya) is associated with the postglacial spread of Magdalenian culture from southwestern European refuges and shares genetic ancestry with the pre-LGM Goyet-Q116-1^3^. The Villabruna genetic cluster (14–7 kya) was found to be the predominant ancestry cluster among Western and Central European hunter-gatherers and is associated with the Azilian, Epipaleolithic, Epigravettian and Mesolithic cultures in Europe^3^. Nevertheless, a more complete picture of the post-LGM population history of western Eurasia remains elusive, as fossils from Southern Europe are still underrepresented in genomic studies.

Meanwhile, recent investigations have shown that dental calculus, a complex and calcified bacterial biofilm formed from dental plaque, saliva and gingival crevicular fluid, is rich in aDNA and proteins^5^. Accordingly, calculus is particularly valuable for characterising diet, oral microbiome and oral disease in ancient populations^5^. Diet is one of the most important lifestyle factors determining human health, playing a pivotal role in shaping the composition of oral microbiomes. Changes in the human diet have implications on the evolution and ecology of the oral microbiome^6^, which in turn can affect gene expression in the immune response system^7,8^. Ultimately, a deeper knowledge of diet and nutrition becomes necessary to solve the complex co-evolution of oral microbiomes and their human hosts.

Dietary information has so far mostly been obtained by stable isotope analysis, which cannot identify the animal and plant species used as diet resources^9^. To overcome this limitation and better characterise the ancient diet and oral microbiomes, dental calculus should be analysed by a double approach to achieve paleogenomics and palaeoproteomic profiling. These methods are complementary in the identification of species characterising the oral microbiome and those consumed for nutrition. Furthermore, the proteomic analysis allows for the reconstruction of both the pathogenic action and the immune response ongoing between an oral microbe and its host, based on the detection of process-specific protein profiles^10^. The combined analysis of different ancient biomolecules can therefore provide a wider perspective and answer complex biological questions about past humans^11^.

Here, we combine analyses of human aDNA from petrous bones, with microbial aDNA and food source ancient proteins from dental calculus, isolated from two Late Upper Palaeolithic (Late Epigravettian) hunter-gatherer individuals from San Teodoro cave (Sicily, Italy; Supplementary Fig. 1, Supplementary Note 1, Supplementary Data 1), dated to 15,322–14,432 cal. BP (Supplementary Note 2, Supplementary Fig. 2, Supplementary Data 3). We used these analyses to investigate genetic ancestry and dispersal of southern European hunter-gatherers after the LGM, and to reconstruct their oral microbiomes and dietary lifestyle. Given the complexity of its archaeological records, Southern Italy is one of the key geographic areas for understanding human and biological responses to postglacial climate evolution in Europe.

## Results

### San Teodoro human data generation and analysis

We extracted DNA from the petrous bones of two Palaeolithic individuals from San Teodoro (Sicily, Italy), and sequenced the obtained libraries to 0.5X (San Teodoro 3—ST3) and 0.1X (San Teodoro 5—ST5) average genomic coverage using shotgun sequencing (Supplementary Data 5). Both samples showed deamination pattern and read lengths typically found in ancient remains^12^ (Supplementary Fig. 3a), and low rates of contamination with modern human DNA (ST3: 0.4% mitochondrial DNA (mtDNA) and 2.3% X-chromosome; ST5: 1.4% mtDNA; Supplementary Data 5, Supplementary Fig. 3b), supporting the authenticity of the generated data.

Genetic sex determination using the fraction of reads mapping to X and Y chromosomes showed that San Teodoro 3 was male while San Teodoro 5 was female, in agreement with the morphological results (Supplementary Data 3–5, Supplementary Note 3, Supplementary Figs. 4–6). Analysis of kinship indicated that the two individuals were not closely related (Supplementary Data 6). The mitochondrial haplogroup of both individuals is U5b2b (Supplementary Data 5), one of the most common mitochondrial haplogroups found in post-LGM hunter-gatherers from Europe^13–15^, and likely associated with the resettlement of Europe after the LGM^3^. The San Teodoro individuals belong to the same subclade as the 14,180–13,780-year-old Palaeolithic (Late Epigravettian) individual Villabruna from Northern Italy, which represents the most diverged haplotype (Supplementary Fig. 7, Supplementary Data 7). Analysis of divergence times estimated the age of haplogroup U5b2b to be around 23 kya (from 19,137 to 27,984), in accordance with previous results^15^. Furthermore, the San Teodoro lineage is closely related with the Paglicci 71 lineage (around 18 Kya^14^) from Apulia (Italy), which refers to a previous phase of the Epigravettian (Evolved Epigravettian).

The Y-chromosome haplogroup determined for San Teodoro 3 is I2a2 (Supplementary Data 5, 8 and Supplementary Fig. 8), a subclade of haplogroup I2 which is common among European hunter-gatherers, and possibly originated in southern Europe during the LGM before becoming widespread in Europe during the Neolithic^16,17^ and today is the most common haplogroup in Balkan peninsula^18^.

### Genetic structure of European Hunter-Gatherers

**Fig. 1:**
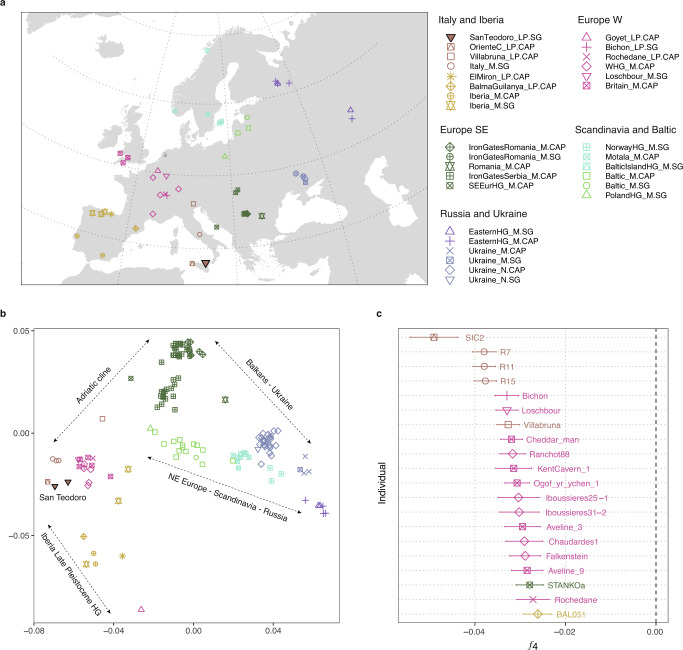
Genetic clustering of European post-LGM hunter-gatherers. **a** Geographic location of new individuals from San Teodoro and previously published post-LGM hunter-gatherer individuals; **b** Multidimensional scaling (MDS) of 138 hunter-gatherer individuals (>50,000 SNPs covered) based on a pairwise identity-by-state (IBS) allele sharing. Major ancestry clines are indicated with dotted arrows. **c** Genetic affinities with the San Teodoro individuals. Shown are point estimates and standard errors for statistic *f*_4_(Mbuti, Test; SanTeodoro_LP.SG, DevilsCave_N.SG), which measures allele sharing of a test individual with San Teodoro compared to Neolithic hunter-gatherers from East Asia. More negative statistics indicate higher genetic affinity with San Teodoro. The top 20 individuals with the highest affinity are shown (Supplementary Data 9).

For the purpose of analysing the genetic diversity of European hunter-gatherers after the LGM, we merged the data from San Teodoro with a panel of previously published hunter-gatherer individuals (Palaeolithic and Mesolithic) from across Europe (Fig. 1a). To visualise the genetic structure of the ancient individuals, we performed multidimensional scaling on a distance matrix derived from pairwise identity-by-state (IBS) sharing of alleles among individuals (Fig. 1b, Supplementary Fig. 9). The two San Teodoro samples fell broadly within the genetic diversity of individuals from western and southern Europe, previously termed Western hunter-gatherers (WHG)^19^ (Fig. 1b, Supplementary Figs. 9–10). Within this group of individuals, San Teodoro clustered most closely with another Late Epigravettian individual from Sicily, Oriente C, and with the Late Upper Palaeolithic/Mesolithic individuals from Grotta Continenza (central Italy), indicating a common gene pool for post-LGM hunter-gatherers from the Italian peninsula from ~14,000 years ago onwards.

The clustering results are also confirmed by *f*_4_-statistics of the form *f*_4_(Mbuti, Test; SanTeodoro_LP.SG, DevilsCave_N.SG), which measures the excess of genetic drift a test individual shares with San Teodoro (from here on merged into a single population due to their high genetic similarity) compared to an outgroup (East Asian hunter-gatherers from Devil’s Gate cave). Results showed that WHG individuals, in particular those from the Italian peninsula, shared the highest amounts of genetic drift with San Teodoro. The highest affinity was observed for Oriente C (SIC2), an individual related to the same cultural sphere of San Teodoro (Late Epigravettian), with a similar age from the same geographic region (Sicily)^20^ (Fig. 1c, Supplementary Data 9).

Using *f*_4_-statistics of the form *f*_4_(Mbuti, Test; HG Italy, San_Teodoro_LP.SG), we next tested whether San Teodoro forms a clade with Italian hunter-gatherer groups, consisting of both Late Upper Palaeolithic (Oriente C, and Villabruna) and Late Upper Palaeolithic/Mesolithic (Continenza), to the exclusion of other test groups. While a clade-like relationship cannot be rejected for the Sicilian hunter-gatherer from Oriente C, significant statistics were observed in tests with the other two groups (Supplementary Fig. 11, Supplementary Data 10). For the central Italian individuals from Continenza, most post-LGM individuals showed significantly increased shared genetic drift compared to San Teodoro. The consistent magnitude of the statistics across individuals with a wide geographic distribution, and chrono-cultural differentiation, suggests possible gene flow between the ancestors of most post-LGM Europeans and Continenza, after their divergence from San Teodoro. Interestingly, the only individual with evidence for higher affinity with San Teodoro than with Continenza was the ~33,000-year-old pre-LGM individual Paglicci133 from Apulia in southern Italy (*f*_4_(Mbuti, Paglicci133; Italy_M.SG, San_Teodoro_LP.SG) = 0.003, Z = 2.6; Supplementary Figs. 9, 11). Paglicci 133 refers to Gravettian which is considered the cultural root (the Gravettian) from which the San Teodoro one (the Epigravettian) was derived in the following several millennia. This suggests possible gene flow involving ancestry related to pre-LGM hunter-gatherers in southern Italy, as previously observed on the Iberian Peninsula^19^, as a possible alternative explanation. A clade-like relationship was also rejected for the relationship of San Teodoro and the ~14,000-year-old northern Italian individual from Villabruna. In contrast to the results obtained with Continenza, in this analysis the individuals from western Europe mostly shared more genetic drift with San Teodoro, whereas individuals from eastern Europe shared more genetic drift with Villabruna (Supplementary Fig. 11, Supplementary Data 10). These results were also reflected in the genetic clustering, where Villabruna was shifted away from the Italian and west European individuals on a cline towards individuals from the Balkans (Iron Gates, Fig. 1b), suggesting further substructure among individuals of the Villabruna cluster.

The genetic diversity of European post-LGM hunter-gatherers has previously been described in terms of a west-to-east cline anchored by two major ancestry groups of western hunter-gatherers (WHG) and eastern hunter-gatherers (EHG)^16^, with some contributions from late Pleistocene hunter-gatherer ancestry in the Iberian Peninsula^21^. In the MDS in Fig. 1b, individuals were maximally differentiated in four distinct ancestry clusters, namely: (i) hunter-gatherers from the Italian peninsula (including San Teodoro), (ii) late Pleistocene hunter-gatherer ancestry (represented by Goyet Q-2), (iii) individuals from the Balkans (Iron Gates) and (iv) EHG from Russia. The remaining individuals were aligned across a number of distinct genetic clines, broadly related to their geographical location, including (Fig. 1b): (i) an Adriatic cline, linking the Italian peninsula to the Balkans; (ii) an Iberia late Pleistocene HG cline of Goyet Q-2 related ancestry in individuals from the Iberian peninsula; (iii) a Balkans-Ukraine cline and (iv) a NE Europe—Scandinavia—Russia cline, linking individuals from the Baltic to Scandinavia and EHG. Furthermore, we found evidence for temporal genetic structure within geographic regions. This was most notably at Iron Gates, where the earlier individuals from Serbia (before 9000 BP), together with the neighbouring individuals from Romania, form one of the four most differentiated clusters (Fig. 1b), whereas later individuals from Serbia (after 9000 BP) were shifted towards those from north-eastern Europe and the Baltics (Fig. 1b, Supplementary Fig. 12).

**Fig. 2:**
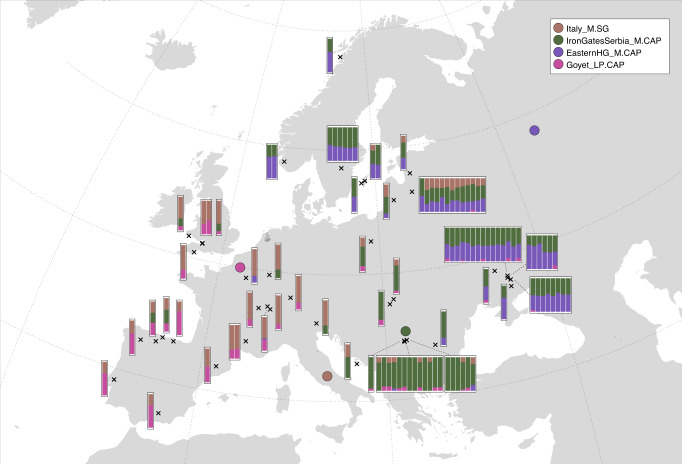
Spatial distribution of hunter-gatherer ancestry clusters. Map showing geographic locations (black crosses) and ancestry proportions (bar plots) of post-LGM hunter-gatherers, inferred using *qpAdm* (Supplementary Data 14). Individuals were modelled using four source groups, representing major post-LGM lineages identified in this and previous studies. Approximate geographic locations of source groups are indicated with coloured symbols.

Motivated by these observations, we used *qpAdm* to infer ancestry proportions of European hunter-gatherers in a four-way model using representatives of the maximally differentiated genetic clusters as sources. The results recapitulated many of the features observed in the genetic clustering. In individuals from western Europe, ancestry related to Italian hunter-gatherers predominated, with varying contributions of late Pleistocene hunter-gatherer (Goyet Q-2) ancestry (Fig. 2, Supplementary Fig. 13, Supplementary Data 11–14). Late Pleistocene ancestry peaked in the Iberian Peninsula, with inferred ancestry proportions as high as 71% (El Miron), consistent with previous results^21^. An apparent influx of Balkan hunter-gatherer related ancestry was observed in the two most recent individuals from northern Iberia (La Brana, Los Canes), suggesting gene flow between distinct ancestry groups towards the end of the hunter-gatherer dominion in western Europe^22^. In eastern Europe, ancestry related to Balkan hunter-gatherers was most abundant, with additional contributions of Italian or EHG ancestry, in line with the geographic origin of the individuals. Genetic continuity could be rejected for many of the later Balkan hunter-gatherers from Iron Gates, which showed evidence for admixture with groups harbouring both Italian- and Goyet Q-2-related ancestry (Supplementary Fig. 13; Supplementary Data 11–14). Finally, EHG-related ancestry was found at highest proportions in individuals from Ukraine, as well as Scandinavian hunter-gatherers^23^. Once again, we observed temporal stratification in ancestry suggestive of local genetic transformations, with an increase in Balkan hunter-gatherer related ancestry in Ukrainian individuals after 10,000 BP^16^ (Supplementary Fig. 13). Taken together, these results document previously underappreciated complexity in the fine-scale genetic structure of post-LGM hunter-gatherers in Europe.

### Diversity of the oral microbiome

**Fig. 3:**
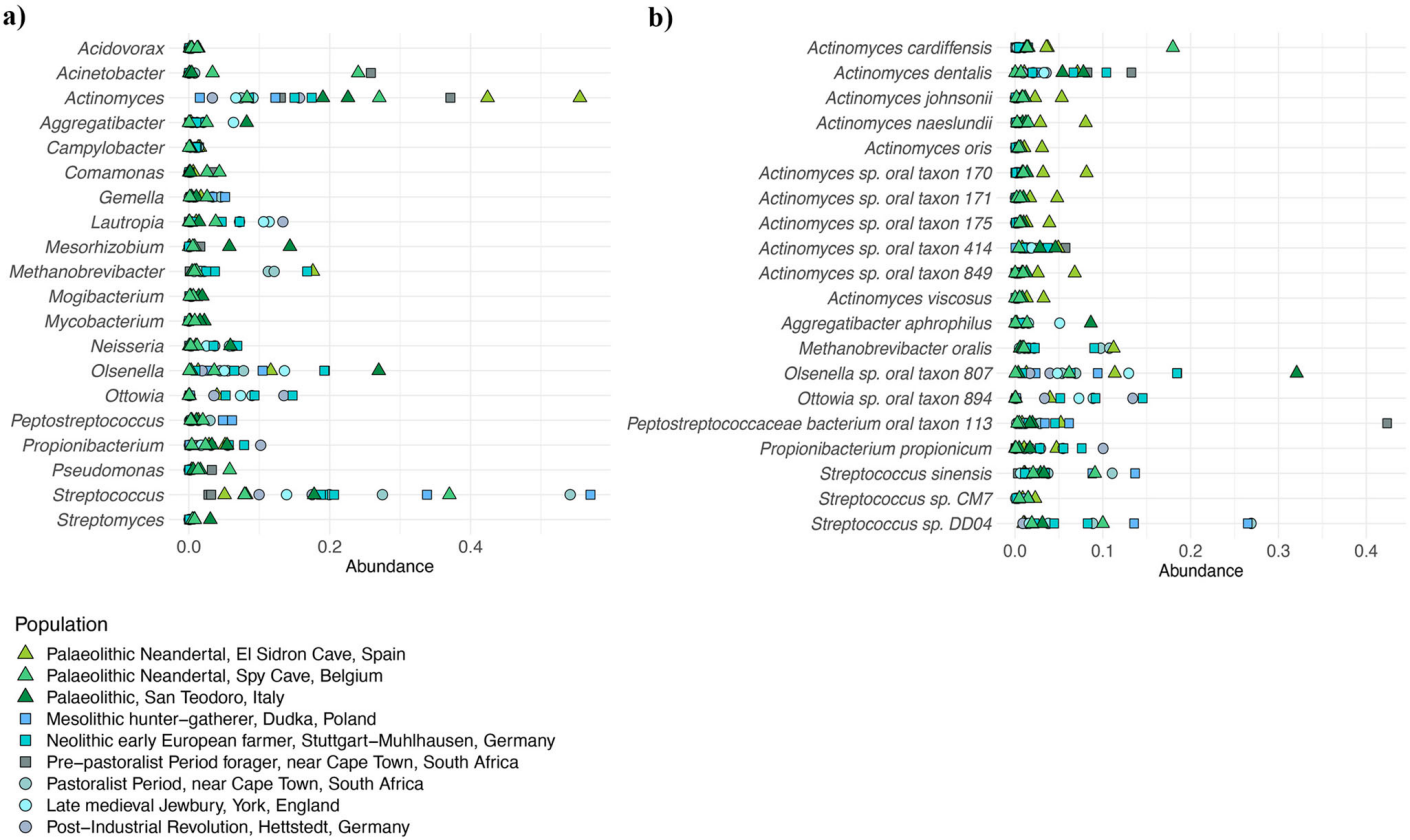
Metagenomic composition of ancient dental calculus. Shown are estimated abundances of the top 20 most abundant genera (**a**) or species (**b**) across all ancient calculus samples (Supplementary Data 18–19).

We sequenced ancient DNA extracted from dental calculus to characterise the oral microbial communities of the two Upper Palaeolithic hunter-gatherers. We obtained a total of 28,732,940 (San Teodoro 3) and 32,249,586 (San Teodoro 5) sequencing reads, which were subjected to metagenomic classification and abundance estimation using Kraken^24^/Bracken^25^ and KrakenUniq^26^, resulting in a final dataset containing 1,639,575 (5.71%; San Teodoro 3) and 2,431,172 (7.54%; San Teodoro 5) classified reads. We first characterised the broad microbial composition of the two San Teodoro individuals (Supplementary Data 15). Both were abundant in *Actinomyces, Streptococcus* and *Propionibacterium*, genera typically associated with the oral microbiome (Fig. 3, Supplementary Figs. 14–16, Supplementary Data 15). San Teodoro 3 was also abundant in *Olsenella*, known to cause endodontic infections, while San Teodoro 5 was abundant in *Aggregatibacter* and *Neisseria*, both a part of the normal oral microbiome. To authenticate sequences of putative ancient microbial origin, we mapped sequencing reads classified at the species level back to their respective reference sequence, and determined aDNA damage profiles, genomic coverage and read edit distance distributions. We find that sequences classified from species associated with the oral microbiome are characterised by high rates of DNA damage, low edit distances and even genomic coverage, strongly supporting their authenticity (Supplementary Figs. 17–20; Supplementary Data 15). In both samples, some species show few or no reads mapping back to their reference genomes, despite high abundance estimated from Kraken/Bracken. However, those likely false positives derive from genera such as *Mesorhizobium*, *Sphingomonas* and *Methylosinus* which are not typical for oral microbiota (Supplementary Data 15).

We analysed the ancient microbiome data from San Teodoro in the context of a reference dataset that comprised a total of 1400 modern samples covering eight oral sites and four other human microbiomes, 75 soil samples (Supplementary Data 16), as well as 17 previously published ancient calculus samples^27^ (Supplementary Data 17). Microbial genera and species associated with oral microbiota were found highly abundant across all ancient samples (Fig. 3, Supplementary Data 18–19), in concordance with the results from San Teodoro. Environmental contamination from soil microbiomes estimated using SourceTracker2^28,29^ was low (<5%) across all ancient calculus samples (Supplementary Fig. 21). To further minimise possible impacts from environmental contamination or false positive classification, we restricted all compositional analyses to sets of microbial species with a minimum of 1,000 unique kmers classified in five or more modern samples, using KrakenUniq^26^. For analyses involving all modern and ancient samples (species set “all”), microbial species identified in all modern metagenomes were included; for analyses restricted to the oral microbiomes (species set “oral”), only microbial species identified in modern samples from oral sites were included (Supplementary Data 20; Supplementary Fig. 22).

**Fig. 4:**
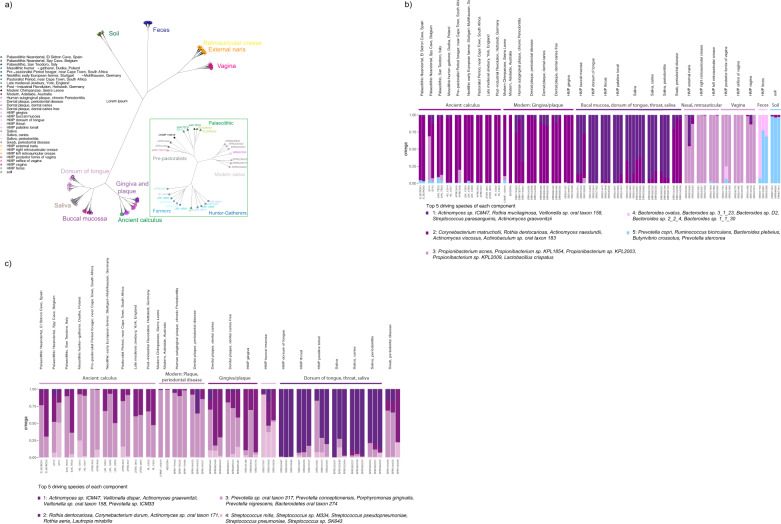
Clustering of ancient microbiomes. **a** Dendrogram showing clustering of microbial composition data using centred log-ratio (CLR) transformed counts and Aitchinson distance, using species set ‘all’. Green inset shows structure among ancient samples only. **b** Bar plots showing Grade of Membership (GoM) for ancient calculus and selected modern samples for k = 5 components, using species set ‘all’ (Supplementary Data 23). **c** GoM of oral samples for k = 4 components, using the species set ‘oral’ (Supplementary Data 23).

We investigated differences in microbial composition using both unsupervised (Principal Component Analysis (PCA), clustering) and supervised (Discriminant analysis of principal components (DAPC)) methods (Supplementary Figs. 23–30). All methods showed samples broadly clustering into communities according to their site of isolation (Fig. 4a; Supplementary Figs. 23–32). Samples from the retroauricular crease and the external naris clustered together, with some overlap of vaginal samples, consistent with previous results^30^. The ancient calculus samples clustered nearest to the plaque or the gingiva samples and were broadly divided according to mode of subsistence: Palaeolithic foragers, hunter-gatherers and farmers (Fig. 4a; Supplementary Figs. 23–32). Soil samples showed a clearly distinct clustering from ancient calculus, consistent with low contamination estimated from SourceTracker in the latter (Fig. 4a; Supplementary Fig. 21). In the PCA, the more recent samples from the introduction of farming and forward were placed nearer to the modern samples than the Palaeolithic and pre-pastoralists (Supplementary Figs. 23–26) and one of the post-Industrialisation samples (IR_13234) clustered with modern samples. Allowing K-means to decide the groupings of all samples before DAPC, all ancient samples were found to cluster together. An exception was one of the Neandertal individuals from Spy cave (SPY1), which clustered with samples from retroauricular crease and the external naris and has previously been found to be contaminated with modern DNA^30^. Modern samples are largely clustered according to their isolation source, though split into smaller groups (Supplementary Data 21). When analysing only the oral samples and corresponding species list, we observed further finer-scale structure. Groups identified by K-means separated all Palaeolithic samples and one pre-pastoralist forager from later hunter-gatherer, Neolithic and post-Neolithic samples (Supplementary Figs. 29–30 and Supplementary Data 22). Except for one ancient sample from the Post-Industrial Revolution period (IR_13234) which clustered with modern oral samples, both clusters of ancient samples were clearly distinct from the modern microbiomes. Restricting to species identified in modern oral samples also diminished the influence of contamination in SPY1, which clustered together with the other Palaeolithic individuals in this analysis.

To further investigate the microbial species distinguishing the different microbiomes and between ancient and modern samples, we performed a Grade of Membership (GoM) analysis^31^. The analysis was run with two to nine components (k = 2 to k = 9), with both all human microbiome samples and only oral samples (Fig. 4b, c, Supplementary Figs. 33–34). In the full microbiome analysis using two components, samples from oral sites and faeces were separated from those of the external naris, retroauricular crease and vaginal sites. Among the ancient samples, SPY1 showed a substantial fraction of its GoM from a component maximised in samples from the external naris, retroauricular crease, consistent with the aforementioned contamination with non-oral microbial communities^27^. At k = 3, vaginal sites were separated from retroauricular creases and external naris, which are characterised with species of the genus *Propionibacterium* commonly associated with skin microbiome^32^. A distinction between oral sites was first observed at k = 5, with the ancient calculus samples more similar to gingiva and plaque, consistent with their sampling location (Fig. 4b). Higher numbers of components revealed additional substructure within microenvironments of the same body site, such as the buccal mucosa at k = 7 which was associated with species of the genus *Streptococcus* such as *Streptococcus mitis* and *Streptococcus pneumoniae*. At k = 9, we observed a division between healthy and periodontal gingiva or plaque samples, associated with *Prevotella* sp. and *Porphyromonas gingivalis* as the driving species for periodontal samples (Fig. 4b, Supplementary Data 23, Supplementary Fig. 33). Restricting to species found in the oral microbiome only, we found that three components (k = 3) separated the different oral compartments of gingiva/plaque, buccal mucosa and dorsum of tongue and saliva. The ancient calculus samples were similar to modern gingival and plaque samples, and their component was associated with species such as *Corynebacterium matruchotii, Propionibacterium propionicum* and *Actinomyces naeslundii*. At k = 4, healthy and periodontal gingiva/plaque samples were distinguished. Clusters with high proportions in healthy samples were associated with species such as *Rothia dentocariosa, Corynebacterium durum, Rothia aeria* and *Lautropia mirabilis*. Samples with periodontal disease on the other hand were characterised by a component including gram-negative, anaerobic bacteria known to show increased abundance with periodontal disease^33^, such as *Prevotella conceptionensis, Porphyromonas gingivalis* and *Prevotella nigrescens*. Interestingly, most of the ancient calculus samples were also highly abundant in the periodontal component (Fig. 4c, Supplementary Fig. 34).

We further characterised differential microbial abundance among the samples using ALDEx2, a method developed for the analysis of compositional data^34,35^. Applying the method to our dataset, we found species including *Methanobrevibacter oralis*, *M. smithii, Olsenella sp. Oral taxon 807* and *Peptostreptococcaceae bacterium oral taxon 113* among those with significantly higher abundance in the ancient samples (Supplementary Data 24–27, Supplementary Figs. 35–36). On the other hand, species of the genera *Veillonella, Streptococcus, Haemophilus, Rothia, Porphyromonas, Campylobacter* and *Neisseria* were significantly more abundant in the modern samples. We found that gingiva/plaque sites were overall depleted in *Streptococcus* species, particularly in samples from individuals with periodontal disease*. Capnocytophaga* species were more abundant in gingiva/plaque samples, whereas *Porphyromonas gingivalis* and *Treponema denticola* were more abundant in samples with periodontal disease and ancient calculus. Interestingly, *T. vincentii* and *Prevotella* species were also more abundant in modern periodontal samples, but not in ancient samples. We did not find any significant differentiation between pre- and post-Neolithic ancient samples (Supplementary Data 28).

### Identification of dietary and human proteins in dental calculus

**Fig. 5:**
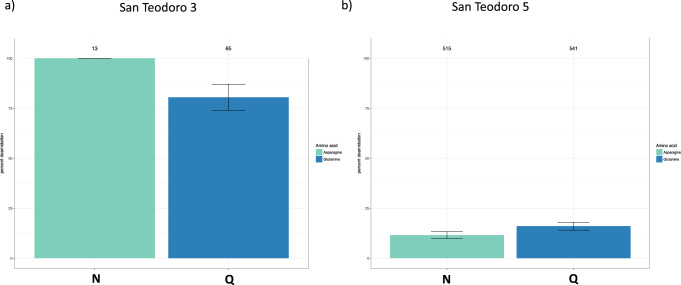
Proteins deamidation. Overall percentage of deamidation for asparagine (N) and glutamine (Q) amino acids for the proteins found in the dental calculus samples: **a** San Teodoro 3; **b** San Teodoro 5. Numbers above each bar represent the number of peptides used for the analysis and the error bars represent standard deviation.

We used palaeoproteomics to identify dietary and human proteins present in the dental calculus of the San Teodoro individuals (Supplementary Data 29–31). To support the endogenous origin of the identified proteins, we calculated the rate of deamidation for asparagine and glutamine, a spontaneous form of hydrolytic damage consistently observed in ancient samples^36^. Both the samples show an advanced rate of deamidation, compatible with the authenticity of the generated data (Fig. 5). The deamidation pattern was also evaluated on the collagen extracted from the petrous bones of the two San Teodoro samples to verify and confirm different preservation states between them (Supplementary Note 4, Supplementary Figs. 37–38).

To avoid spectra misinterpretation, we included all common modifications that could affect ancient proteins, such as deamidation, as variable modifications in the analyses (see materials and methods) and all the spectra associated has been manually inspected, validated and annotated. Compared to San Teodoro 3, San Teodoro 5 shows a lower rate of damage and a higher number of confidently identified peptides and proteins, suggesting a different state of protein preservation for the two San Teodoro dental calculus samples (Fig. 5, Supplementary Data 29–31). In San Teodoro 3, the human proteins identified are limited mostly to collagen^37^. In San Teodoro 5, a total of 22 human proteins are observed (Supplementary Data 30), most of which were associated with immune response, as previously observed^10^.

The food proteins provide an unprecedentedly detailed insight into the diet of the San Teodoro individuals. While stable isotope analyses previously could only suggest a relevant consumption of animal proteins, typical of hunter-gatherers^38^, palaeoproteomic analysis identified some of the plant and animal taxa each individual consumed (Supplementary Data 29, 31).

Specifically, in both calculus samples from San Teodoro 3 and 5 (Supplementary Data 29, 31) we identified bovine Collagen Type I Alpha 1 and 2, arguably from aurochs (*Bos primigenius*), the only bovine species known to live in Sicily during the Late Upper Palaeolithic. This observation was also supported by the recovery of DNA sequences originating from the genus *Bos* in the metagenomic data of San Teodoro 3 (Supplementary Data 32). For similar reasons, collagen proteins found in San Teodoro 5 and confidently assigned to pig (*Sus scrofa/Sus scrofa domesticus*) and equine (*Equus caballus/Equus asinus*) should be attributed to the wild species *Sus scrofa* and *Equus hydruntinus* (Supplementary Data 29, 31). Our results therefore conclusively support the consumption of aurochs, wild boar and European wild ass, as previously suggested by the recovery of bone remains from these taxa in the faunal record of many Late Epigravettian Sicilian deposits^39^ including San Teodoro cave (Supplementary Note 2).

The palaeoproteomic results also showed the consumption of fish in both samples (Supplementary Data 29, 31). In San Teodoro 3, only one freshwater fish taxon, common carp (*Cyprinus carpio*), was identified, while in San Teodoro 5, both freshwater and marine fishes were found: *Cyprinus carpio* and the small, shallow-water shark *Scyliorhinus canicula*. We caution the peptides recovered could also match other close taxa, whose proteome sequences are not yet publicly available. While cyprinids are frequently recorded in Western European Upper Palaeolithic deposits, including Epigravettian sites in continental Italy^40^, no evidence of this group has been previously reported from Palaeolithic deposits in Sicily. The presence of freshwater and marine fish in the diet of the two individuals from the San Teodoro cave is fully coherent with its geographic position (a few hundred metres away from the streams Inganno and Furiano, and from the marine coast, Supplementary Fig. 1) and in accordance with the archaeological record know in Sicily and the Mediterranean area for the Upper Palaeolithic^41–43^.

In San Teodoro 5 six seed storage proteins match modern chickpea (*Cicer arietinum*) (Supplementary Data 29, 31). Chickpea is one of the crops present in primaeval agriculture in the Near East and Europe. The first archaeological findings of this species are associated with the Pre-pottery Neolithic site (El-Hemmeh) in Jordan dated back 11,100–10,610 BP^44^. From the Near East, the chickpea spread to south-eastern Europe with the Neolithic transition^44^. There is no evidence of chickpea consumption during the Upper Palaeolithic in southern Europe. At the end of the Iron Age in the Mediterranean regions, the main spontaneous pulses were the red pea (*Lathyrus cicera*) and the grass pea (*Lathyrus sativus*). Their consumption was attested in Spain during the Upper Palaeolithic^45^, however so far there is no palaeobotanical evidence of the presence of this group of wild plants in the Sicilian Upper Palaeolithic. At the time our analysis was performed, the plant proteins we identified were not among those whose sequence was known for the genus *Lathyrus*. Based on the archaeological context the samples originate from, we exclude the consumption of chickpeas and consider instead the exploitation of wild pulses highly plausible, possibly of the *Lathyrus* genus. All the plant proteins we identified are seed storage proteins, and their consumption may have occurred after the transformation of the seeds into flour. The use of flours from wild plants is documented in other contexts of the Upper Palaeolithic^46^.

Finally, in San Teodoro 5 we identified Collagen Type I Alpha 1 matching domesticated ovicaprid species: *Capra hircus/Ovis aries/Ovis aries musiman*, none of which is likely to be present in the archaeological context of San Teodoro and in the other Epigravettian sites in Sicily. Mouflon (*Ovis gmelini*), the wild species closest to sheep, is not present in Late Upper Palaeolithic in Southern Europe, while ibex (*Capra ibex*), the wild species closest to goat, is well documented during the Late Glacial in Southern peninsular Italy (the southernmost and closest documentation to Sicily is in the Grotta del Romito in Northern Calabria)^47^ and never inhabited in Sicily^48^. Then we interpret the identification of ovicaprid collagen as a possible evidence of ibex exploitation.

## Discussion

In recent years, rapid progress has been made in the recovery of DNA from ancient human remains. These data have revolutionised our understanding of human demographic history, and the processes that shaped genetic diversity in the past. In this study, we combined ancient genomic, metagenomic and palaeoproteomic data to characterise ancestry, diet and microbial environment of two Upper Palaeolithic hunter-gatherers from Sicily.

The genetic ancestry of the Palaeolithic population of San Teodoro fell broadly within the variation associated with hunter-gatherer individuals from Europe, in particular those of other central-southern Italian Late Upper Palaeolithic/Mesolithic individuals: Oriente C and Grotta Continenza. The high genetic affinity among the Sicilian HGs suggests that a founder effect may have played a key role in determining the genetic makeup of the post-LGM Sicilian population. We observed several genetic clusters and clines in European HGs, associated with the geographical localisation of the individuals studied. Among them, our human genetic results highlight the presence of an Italian genetic cluster which likely played a key role during the post-LGM resettlement of western Europe. These results are in accordance with the contraction of animal and plant species in southern areas of Europe, during the LGM^1^ and the following expansion from these glacial refugia to northern and central areas of Europe^3,13^ from 18 kya BP, thanks to the rapid climatic amelioration. On a continental scale, our results suggest that geographic clines and isolation-by-distance played an important role in shaping European hunter-gatherer diversity. Nevertheless, we also find evidence for local transformations and possible migrations in regions including northern Iberia, the Balkans and Ukraine.

The integration between the metagenomic and the proteomic analysis of dental calculus provides a detailed insight into the diet and the composition of the oral microbiome of the human hunter-gatherers during the Upper Palaeolithic. Specifically, we find, in line with previous results^30^, that ancient calculus microbiomes broadly cluster according to the mode of subsistence, which is also true for these Epigravettian individuals. The oral microbiomes and the several oral animal proteins found in San Teodoro individuals are consistent with the well-founded meat-rich diet hypothesis inferred by the rich archaeological (faunal) record from Late Upper Palaeolithic contexts and further supported by the high nitrogen stable isotope ratio values identified in several Late Upper Palaeolithic individuals from Sicily^38^. This result is also in accordance with the protein data where a lack of plant proteins was observed in the samples analysed. In fact, only the consumption of legume seed storage proteins was identified in San Teodoro 5. It should be noted that the absence of specific food proteins does not necessarily mean that a particular food resource was not regularly ingested. However, the differentiation between the San Teodoro and early farmer oral microbiomes and the lack of plant proteins found in the samples analysed could suggest low plant intake in this Upper Palaeolithic community. Still, this result confirms that the dietary habits of Epigravettian hunter-gatherers included some plant foods, as suggested in other archaeological contexts in Northern and Central Italy^42,49^.

The identification of proteins matching the genus of Capra, which could possibly belong to ibex, opens speculations on the dynamics of human populations of Sicily in the Late Glacial and can contribute to the study of mobility of Upper Palaeolithic hunter-gatherers between the island and the Italian peninsula or some exchange between Calabria and Sicily. So far there is no archaeozoological and palaeontological evidence supporting the presence of ibex in Upper Palaeolithic Sicily^50^, instead, this species was very common on the mountains of nearby Calabria during the Upper Palaeolithic, for instance at the site of Grotta del Romito^47^. The hypothesised consumption of ibex by the San Teodoro 5 individual can either be an indicator of the movement of the San Teodoro 5 individual to (or from) peninsular Italy, even though an indirect supply cannot be ruled out as a consequence of transport of ibex meat from Calabria to Sicily by other Epigravettian hunter-gatherers. Either way, the results of the proteomic analysis provide important insights to advance hypotheses on the mobility of the Sicilian hunter-gatherers and on their contacts with the Italian peninsula. In addition, the individuals of San Teodoro are not the oldest evidence of human colonisation of Sicily, which, on a radiometric basis and in accordance with current archaeological evidence, dates back to about a millennium earlier^51,52^. Therefore, they could be evidence of a further arrival of human groups in Sicily from Calabria where they might have eaten ibex meat. On the other hand, the possibility of occasional or habitual movements towards Southern Calabria by the Epigravettian hunters of San Teodoro, with a short sea-crossing, cannot be ruled out.

The presence of marine and freshwater fish species fits with the framework of knowledge about the exploitation of the aquatic resources in the Mediterranean basin during the Upper Palaeolithic. Sea fish consumption evidence at San Teodoro is in accordance with what is already known for the Late Palaeolithic in Sicily^53^ and in other coeval contexts from the Mediterranean basin. In Sicily, an increase in marine exploitation has been observed in the Mesolithic due to a combination of sea level rising, population growth and terrestrial resource depletion after the LGM^53^. However, only a few Upper Palaeolithic Sicilian sites provide sufficient stratigraphic and chronological information about the exploitation of aquatic resources^53^. Our results further confirm and illuminate the exploitation of marine and freshwater resources during Late Epigravettian, showing the important benefits of the proteomic approach to identify species often absent in the archaeological records of the ancient sites.

In conclusion, while the sample size of two individuals warrants some caution in generalising our findings to a broader context, they nevertheless demonstrate the value of integrating ancient genomic, metagenomic and metaproteomic data in the study of prehistoric hunter-gatherer communities. Using this approach, we could show that the individuals from San Teodoro were part of a likely genetically homogenous Sicilian hunter-gatherer metapopulation, with a mode of subsistence predominantly relying on exploitation of meat and aquatic resources. Applying it to a broader range of prehistoric hunter-gatherer communities in future studies is needed to reach a more comprehensive understanding of their population dynamics and ecology.

## Methods

### Archaeological sample material

Two individuals from San Teodoro cave in Sicily: San Teodoro 3 (male) and 5 (female), were sampled for aDNA analysis and lifestyle evaluation. For the human aDNA analysis the petrous bone was selected.

For the lifestyle evaluation, dental calculus was analysed by mass spectrometry-based proteomics and for the oral microbiome reconstruction by the metagenomic approach.

Dental calculus was carefully removed from the tooth using a sterile periodontal scaler and around 20–30 mg were transferred into DNA LoBind Eppendorf tubes. Between one sampling and another, the periodontal tools were cleaned with bleach (concentration 5%) and after with ethanol (70%)^54^. Each sample was then divided into two different tubes, 22.8 mg (San Teodoro 3) and 9.9 mg (San Teodoro 5) for metagenomics analysis and about 7 mg each for proteomic analysis, 50 mg have been instead used for protein extraction from petrous bones. All the molecular work was performed in DNA clean laboratory facilities at the Lundbeck Foundation GeoGenetics Centre, Globe Institute of the University of Copenhagen.

### DNA extraction

The Allentoft^55^ protocol was used for the aDNA extraction from both ancient matrices. A starting amount of 150–400 mg of bone powder and 10–23 mg of dental calculus were added a pre-digestion buffer and incubated for 45 min at 37 °C in order to remove the surface contaminants. Negative extraction controls were processed along with the samples. After centrifugation at 2000 × *g* for 2 min, the supernatants were discarded and a new digestion buffer was added, then the samples were left for 24 h at 37 °C. The DNA extraction was performed on the digestion buffer by Silica powder. After centrifugation at 2000 × *g* for 2 min, the supernatant was transferred into new tubes and 100 ml of silica suspension and 10× volume of binding buffer was added. This solution was incubated at room temperature for 1 h. Afterwards, the samples were centrifuged at 2000 × *g* for 2 min and the supernatant was discarded. An additional 1 ml of binding buffer was added to the samples and the DNA was cleaned by ice-cold ethanol in a new tube. Finally, the DNA was eluted in 90 μl Qiagen EB buffer.

### NGS library preparation

DNA libraries for sequencing were prepared following a method proposed by Allentoft^55^. This method is divided into 4 steps: End-repair, Quick Ligation, Fill-in and Indexing. In each step, a negative library control was included.

For the End-repair, around 20 μl of DNA extraction was used and NEB End-repair (module E6050L) Mix was added, according to the manufacturer’s instructions, to make a total of 25 μl. The solution was incubated at 12 °C for 20 min and 37 °C for 15 min. Before the ligation a purification step by Qiagen MinElute spin columns was performed and the DNA was eluted in 17 μl EB buffer.

In the Quick Ligation step, Illumina specific adapters^56^ were added to 15 μl of purified DNA by adding the NEB Quick Ligation Mix (module E6056L) and the solution was incubated at 20 °C for 15 min. Then, the mix was purified using Qiagen MinElute spin columns and the DNA was eluted in 20 μl Qiagen EB buffer.

For the Fill-in, 30 μl of NEB (module M0275L) reaction mix was added and incubated at 65 °C for 20 min and 80 °C for 20 min. The library was then quantified using IS8 and IS7 primers and SYBER green solution, according to manufacturer’s instructions. The quantification results were used to assess the optimal number of PCR cycles required for DNA library indexing. The indexing was performed by adding 1 μl of each primer (10 μM, inPE forward primer and indexed reverse primer) and 2× Kapa (following the manufacturer’s temperature instruction). After the indexing, the amplified DNA was purified by Qiagen MinElute Kit, eluted in 50 μl EB buffer, and quantified using an Agilent Bioanalyzer 2100.

The libraries were shotgun sequenced by the Illumina HiSeq 2500 and HiSeq 4000 platforms (81 bp single-read) at the Danish National High-Throughput DNA Sequencing Centre, University of Copenhagen, Denmark. For the run on Illumina HiSeq 4000, the library preparation followed the same steps previously described, but dual-indexed libraries construction and amplification were used.

### Bioinformatics pipeline

AdapterRemoval 1.5.2^57^ was used to remove the Illumina adapter sequences. Reads were mapped against the human reference genome build 37 with BWA 0.6.2^58^. Only reads with above 30 in mapping quality were kept using samtools 0.1.18^59^. Eventually, the PCR duplicate reads were removed by Picard MarkDuplicate http://broadinstitute.github.io/picard/. The mapping statistics for each sample analysed are reported in Supplementary Data 5.

The sex determination was performed by Skoglund and colleagues^60^ python script based on Rg parameter (Rg value lower than 0.016 is consistent with a female while result above 0.077 with a male). The results are reported in Supplementary Data 5.

### DNA authentication

DNA contamination is one of the most serious problems in the aDNA study of samples from museum collections, for this reason, strict approaches were applied. First of all, the deamination pattern in the 5’ and 3’end (C to T transition and G to A, respectively) was assessed by mapDamage 2.0^61^ (Supplementary Fig. 3a) in the results obtained from both the biological matrices.

Moreover, two different methods were also used for the human aDNA authentication: the estimation of contamination in X-chromosome (for male sample) and the evaluation of the probability of mitochondrial DNA contamination (Supplementary Data 5, Supplementary Fig. 3b). The X-chromosome contamination was evaluated just for the male individual (San Teodoro 3) applying ANGSD package^62^ following the commands suggested by the authors.

The mitochondrial contamination was evaluated for both samples by contamMix 1.0^15^ that it reports a Bayesian-based estimate of the posterior probability of the contamination proportion.

### Mitochondrial and Y-chromosome haplogroups assignment

For both samples, the mitochondrial haplogroup was assessed by a command line of haplogrep2^63^ (Supplementary Data 5).

While for San Teodoro 3 (male sample) also Y-chromosome haplogroup was identified. Bcftools mpileup and bcftools call were used for the genotypes call with only bi-allelic SNP sites of the Y-chromosome from the International Society Of Genetic Genealogy (ISOGG, http://www.isogg.org, version 10.107). By epa-ng we built a phylogenetic tree of Y-chromosome sequences from the 1000 Genomes project, with maximum likelihood placement of ST3 (Supplementary Fig. 8).

### BEAST analysis

BEASTv1.8.4^64^ was used to reconstruct the phylogenies of the U5b mitochondrial haplogroup. BEAUti v1.8.4 was applied to generate the input file for BEAST analysis. We used the radiocarbon dates of the ancient samples (Supplementary Data 7) as calibration points in the tree inference, and after analysis with jModelTest 2.1.10^65^ we applied the HKY substitution model^66^ with gamma plus invariant sites and strict clock with a prior of 2.2 10^−8^ µ/site/year^67^. The phylogenetic reconstruction was carried out on 32 ancient samples with MareuilLesMeaux1, haplogroup U5a2^14^, as outgroup. We used the Extended Bayesian Skyline Plot method for mitochondrial and the MCMC chains were run for 10^8^ states and sampled every 10^4^ states. MCMC runs were evaluated using Tracer (v1.6) (http://tree.bio.ed.ac.uk/software/tracer/). The resulting trees were annotated by TreeAnnotator v1.8.4 and visualised by FigTreev1.4.3 (http://tree.bio.ed.ac.uk/software/figtree/).

### Kinship analysis

We also determined the kinship relationship between the San Teodoro individuals inferring rates of identity-by-descent sharing between pairs of individuals by the kinship coefficient estimator implemented in KING^68^. This is based on pairwise identity-by-state (IBS) sharing, obtained estimating the 2D-SFS for each pair using realSFS tool from the ANGSD package^62^. Expected values for the different estimators and selected degrees of relatedness are shown in Supplementary Data 6.

### Ancestry analysis

Analyses of genetic ancestry of the San Teodoro individuals were carried out using a dataset including previously published Eurasian hunter-gatherer individuals (Supplementary Data 33). Genetic similarity measures were estimated based on pairwise sharing of alleles identical-by-state (IBS). Population structure was visualised using multidimensional scaling on a distance matrix calculated as 1- p(IBS). Patterns of admixture and shared genetic drift were evaluated using *f*-statistics, calculated using Admixtools 5.0^69^. Ancestry proportions of post-LGM hunter-gatherers were inferred using *qpAdm*, using a set of 12 outgroups (Mbuti.SG, Yana_UP.SG, UstIshim, Sunghir_UP.SG, IronGatesRomania_M.SG, CHG_M.SG, Botai_EBA.SG, FunadomariJomon_N.SG, Kolyma_River, Zagros_EN.SG, SanTeodoro_LP.SG, EasternHG_M.SG).

### Dental calculus metagenomics analysis

The metagenomic part includes two ancient Upper Palaeolithic Italian calculus samples from San Teodoro. We also included the published calculus samples from Weyrich and colleagues^27^ and for modern comparison, all whole-genome sequenced Human Microbiome Project (HMP)^30^ samples, and six other oral microbiome studies, PRJNA383868, PRJNA230363, PRJNA255922, PRJNA396840, PRJEB14383, PRJEB24090 and soil samples with a known source material. An overview of the included ancient samples is shown in Supplementary Data 17.

All samples were trimmed with AdapterRemoval^57^ with a minimum length of 30 bp and quality above 20 on the phred scale. Metagenomic classification and abundance estimation was carried out using Kraken 1.0^24^ and Bracken^25^, on a database including all bacterial, archaeal, fungal and viral genomes in the NCBI RefSeq database (February 2017), as well as the human genome. Authentication of ancient DNA was performed by mapping reads classified at the species level back to the respective reference sequence using Bowtie2 2.3.2^70^. Ancient DNA damage profiles and fits were obtained using the Bayesian algorithm implemented in metaDMG (https://pypi.org/project/metadmg). For downstream compositional analyses, we used KrakenUniq^26^ to filter for false positives and environmental contaminants of the ancient samples. The data was filtered by a species list including bacteria, fungi and archaea. Only species with above ten reads classified and at least 1000 unique k-mers, or twice as many unique k-mers as reads were included, if present in at least five modern samples. All other analysis was done in R version 3.5.1. For the distance-based methods, the transformation method for compositional data proposed by Gloor et al.^71^ was used, i.e. first a centred log-ratio transformation is applied to the count data and Principal Component Analysis (PCA) with Euclidean distance and dendrograms with Aitchison distance. The data is normalised with the Grade of Membership (GoM)^31^ analysis from the CountClust R package, therefore the raw counts from Bracken were input. ALDEx2^35^ for significant species diversity testing, includes normalisation with Dirichlet distribution using 128 Monte Carlo instances, centred log-ratio transformation and significance testing with both Welch’s *t*-test and Wilcoxon rank test and multiple hypothesis testing with Benjamini and Hochberg false discovery rate. SourceTracker2^28,29^ was used for assessing the proportion of soil contamination. To screen for sequencing reads from likely dietary sources, we used KrakenUniq^26^ on a database of all complete mitochondrial and plastid genomes in the RefSeq database, requiring a minimum of unique 100 kmers classified at the taxonomic level of genus. All plots were plotted with ggplot2^72^.

### Dental calculus and petrous bone protein extraction

The protein extraction was performed following the method proposed by Jersie-Christensen et al.^73^, with a blank extraction control included. To clarify the role of dental calculus in protein preservation we compared the deamidation patterns of proteins obtained from dental calculus and petrous bone of the same individuals. The sample preparation of the bone fragments closely followed that of the dental calculus. The main difference was the overnight demineralisation: for dental calculus 1 ml 15–20% acetic acid was added to about 7 mg of powder while about 50 mg of the bone fragments were demineralised in 300 ml of EDTA pH 8. After centrifugation for 10 min at 2000 × *g* the supernatant was removed, for both sample types. A lysis, reduction and alkylation buffer (2 M guanidine hydrochloride, 10 mM tris(2-carboxyethyl) phosphine hydrochloride, 20 mM chloroacetamide in 100 mM TRIS pH 8,5) was then added to the powder and the pH was adjusted to 7–9. The pellet was crushed by disposable sterile micro-pestles and then incubated either at 99 °C for 10 min (calculus) or at 80  °C for 2 h (bone) at 500 rpm. The protein concentration was then measured by Bradford Assay. The samples were then digested with rLysC (0.2 µg, Promega, Sweden) incubating under agitation at 37 °C for 2–4 h. Subsequently, the samples were diluted to a final concentration of 0.6 M GuHCl solution adding 25 mM Tris in 10% acetonitrile. This was followed by digestion by trypsin (0.8 µg, Promega, Sweden) and incubation overnight at 37 °C under agitation. To stop the digestion, the samples were acidified (pH < 2) using 10% trifluoroacetic acid, then the proteins were collected in home-made C18 StageTips and stored in the freezer until mass spectrometry analysis.

### LC-MS

Dental calculus samples were eluted from the stage tips using 20 μL 40% ACN in water and then 10 μL 60% ACN while the bone samples by 30 μL 40% ACN in water both into a 96-well MS plate. Samples were placed in a SpeedVac^TM^ Concentrator (Thermo Fisher Scientific, Denmark) vacuum centrifuge at 40 °C until approximately 3 μL of the solution was left and then 5 μL of 0.1% TFA, 5% ACN was added.

Samples were then separated on a 15 cm column (75 μm inner diameter) in-house laser pulled and packed with 1.9 μm C18 beads (Dr. Maisch, Germany) on an EASY-nLC 1200 (Proxeon, Odense, Denmark) connected to a Q-Exactive HF (Thermo Scientific, Bremen, Germany) on a 77 min gradient. Five microliters of sample was injected. The column temperature was maintained at 40 °C using an integrated column oven. Buffer A was milliQ water. The peptides were separated with increasing buffer B (80% ACN and 0.1% formic acid), going from 5% to 30% in 50 min, 30% to 45% in 10 min, 45% to 80% in 2 min, held at 80% for 5 min before dropping back down to 5% in 5 min and held for 5 min. The flow rate was 250 nL/min. A wash-blank method using 0.1% TFA, 5% ACN was run in between each sample to hinder cross-contamination.

The Q-Exactive HF was operated in data-dependent top 12 mode (dental calculus) and top 10 mode (bones). Spray voltage was 2 kV, S-lens RF level at 50, and heated capillary at 275 °C. Full scan mass spectra were recorded at a resolution of 120,000 at m/z 200 over the *m/z* range 350–1400 with a target value of 3e^6^ and a maximum injection time of 25 ms. HCD-generated product ions were recorded with a maximum ion injection time set to 45 ms (dental calculus) and 118 ms (bones) with a target value set to 2e^5^ and recorded at a resolution of 30,000 (dental calculus) and of 60,000 (bones). The normalised collision energy was set at 28% and the isolation window was 1.2 m/z with the dynamic exclusion set to 20 s.

### Protein data analysis

Data analysis was performed with MaxQuant version 1.5.3.30^74^ with oxidation (M), Acetyl (protein N-term), deamidation (NQ) and hydroxyproline set as a variable modification and carbamidomethyl (C) as a fixed modification. Digestion enzyme was set to trypsin with a maximum of two missed cleavages allowed. For the identification, a minimum score of modified and unmodified peptides of 40 was used and a Peptide Spectral Match (PSM) and Protein false discovery rate (FDR) of 0.01 cut-off was set. All other parameters were left for the default for orbitrap mass spectrometers. Different databases and several searches were performed on the two biological matrices. First, for dental calculus, the entire SwissProt database (downloaded in January 2017) was used for the first screening and then a search against a FASTA file built using all the proteomes of the species identified by the first search and the human reference proteome from UniProt (downloaded in August 2018) was performed. Likewise, the bone fragments were searched just against the human reference proteome from UniProt. The peptides identified were then filtered applying quality controls. First of all, the resulting proteins group output was filtered to remove reverse and common contaminants. Furthermore, all protein groups with a value of Razor + unique less than two were removed. The spectra for each peptide associated with a proteins group were then manually validated and the species was confirmed by BLAST search^75^. All identifications are based on 100% identity, and common sources of misidentifications (for example, leucine vs isoleucine and deamidated residues) were also checked.

Finally, we calculated the asparagine and glutamine deamidation rate using the python tool proposed by Mackie et al.^36^ in order to assess molecular damage associated with the antiquity of the remains. The deamidation rate was evaluated on all reported proteins in Supplementary Data 31 and also separately on collagen proteins identified in dental calculus and bone samples (Supplementary Note 4).

### Statistics and reproducibility

Patterns of human admixture and shared genetic drift were evaluated by *f*-statistics using a reference panel of 170 individuals. Human Microbiome project, 6 other oral microbiome studies and 21 ancient human dental calculus genomes already published, have been used for the metagenomic comparison. The transformation method for compositional data was used to analyse the samples. Detailed information of the statistical analyses carried out as described in the methods section. All analyses can be reproduced by accessing the associated data linked in the Data Availability statement.

### Reporting summary

Further information on research design is available in the Nature Portfolio Reporting Summary linked to this article.

## Supplementary information

Supplementary Information

Description of Additional Supplementary Files

Supplementary Data 1-33

Reporting Summary

## Data Availability

Source data underlying Fig. 1c are reported in Supplementary Data 9; Fig. 2 in Supplementary Data 14; Fig. 3 in Supplementary Data 18–19 and Fig. 4b, c are presented in Supplementary Data 23. The palaeontological remains are in the Museo e Istituto Fiorentino di Preistoria, Florence (Italy) with the following accession numbers: for San Teodoro 3 ST3 for San Teodoro 5 ST5. Raw genetic sequencing data are available at the European Nucleotide Archive with the accession number PRJEB55789. The mass spectrometry proteomics data have been deposited to the ProteomeXchange Consortium via the PRIDE^76^ partner repository with the dataset identifier PXD024346.
