# Supplementary material for: Genomic ancestry, diet and microbiomes of Upper Palaeolithic hunter-gatherers from San Teodoro cave
Source: Commun Biol. 2022 Nov 18;5:1262. doi: 10.1038/s42003-022-04190-2 (PMC9674856; doi:10.1038/s42003-022-04190-2)
Supplement: Supplementary file 2 — Description of Additional Supplementary Files [file 42003_2022_4190_MOESM2_ESM.pdf]

Description of Additional Supplementary Files

File Name: Supplementary Data 1 to 33:

Description:
Supplementary Data 1: List of the human samples from San Teodoro cave.

Supplementary Data 2: AMS Radiocarbon dates on human samples from San Teodoro: San Teodoro 1 already published and the new radiocarbon dates on San Teodoro 4 and San Teodoro 5 presented in this study.

Supplementary Data 3: Postcranial bone measurements recordable in San Teodoro 1; 3; 4; 5 in comparison to the average values of the Upper Palaeolithic period.

Supplementary Data 4: Cranial morphological features commonly used for sex determination.

Supplementary Data 5: Sequencing data details for the two newly reported samples (San Teodoro 3 and San Teodoro 5).

Supplementary Data 6: Results of kinship analysis between the San Teodoro samples by identity-by-descent-based relatedness estimates.

Supplementary Data 7: Information of the ancient individuals used for the phylogenetic tree reconstruction of the mitochondrial haplogroup U5b.

Supplementary Data 8: Y chromosome haplogroup I SNPs identified in San Teodoro 3.

Supplementary Data 9: Results of the f4 statistics on the form f4(Mbuti,Test; SanTeodoro_LP.SG, DevilsCave_N.SG) used to produce Fig. 1c.

Supplementary Data 10: Results of the f4 statistics on the form f4(Mbuti, Y; X, San_Teodoro_LP.SG) used to produce Supplementary Figure 11.

Supplementary Data 11-14: Results of the qpAdm analyses from 1 to 4 sources groups used to produce the Fig. 2 and Supplementary Figure 13.

Supplementary Data 15: Bracken estimates and mapping results of the top 50 species in San Teodoro 3 and San Teodoro 5 used to produce the Supplementary Figure 17-20.

Supplementary Data 16: Sediment dataset used for dental calculus contamination test.

Supplementary Data 17: Ancient samples used for dental calculus metagenomics comparison.

Supplementary Data 18-19: relative abundances of oral genera (18) and species (19) across all ancient calculus samples, data used to produce the Figure 3.

Supplementary Data 20: Species included in filtered species lists “all” and “oral”.

Supplementary Data 21-22: DAPC all microbiomes groups by K-means used to produce the Supplementary Figure 27-30.

Supplementary Data 23: Grade of Membership results used for the Figure 4b.

Supplementary Data 24-27: All ALDEx2 results for oral samples and for gingiva/plaque samples used to produce the Supplementary Figure 35-36.

Supplementary Data 28: All ALDEx2 results for ancient samples.

Supplementary Data 29-31: Dental calculus proteins results: 29 Dietary proteins; 30 Human proteins; 31 Observed genus-diagnostic peptides for the identified food proteins.

Supplementary Data 32: KrakenUniq San Teodoro 3 results for food sources.

Supplementary Data 33: Information about the ancient individuals dataset used for the human genetic analysis.
